# Mutation profile of primary subungual melanomas in Caucasians

**DOI:** 10.18632/oncotarget.27642

**Published:** 2020-06-23

**Authors:** Aneta Borkowska, Anna Szumera-Ciećkiewicz, Mateusz Spałek, Paweł Teterycz, Anna Czarnecka, Artur Kowalik, Piotr Rutkowski

**Affiliations:** ^1^Department of Soft Tissue/Bone Sarcoma and Melanoma, Maria Sklodowska-Curie National Research Institute of Oncology, Warsaw, Poland; ^2^Department of Pathology and Laboratory Medicine, Maria Sklodowska-Curie National Research Institute of Oncology, Warsaw, Poland; ^3^Diagnostic Hematology Department, Institute of Hematology and Transfusion Medicine, Warsaw, Poland; ^4^Department of Experimental Pharmacology, Mossakowski Medical Research Centre, Polish Academy of Sciences, Warsaw, Poland; ^5^Department of Molecular Diagnostics, Holy Cross Cancer Centre, Kielce, Poland; ^6^Division of Medical Biology, Institute of Biology, Jan Kochanowski University, Kielce, Poland

**Keywords:** melanoma, acral melanoma, subungual melanoma, nail apparatus melanoma, *SMAD4*

## Abstract

Background: Specific genomic profile of cutaneous melanomas is related to UVR exposure, which exerts biological and therapeutic impact. Subungual melanoma (SUM) is an exceedingly rare disease; therefore, it is not well characterized. SUM pathogenesis is not related to UVR induced DNA damage and expected to differ from other melanoma subtypes. Our study aimed to define the mutation profile of SUM in Caucasians.

Materials and Methods: Next-generation sequencing-based genomic analysis was used to identify frequently mutated loci in 50 cancer-related genes in 31 SUM primary tumors.

Results: The most abundant mutations in SUM were found in *KIT* – in 13% of cases and *NRAS* – also in 13%, while *BRAF* - only in 3% of cases.

Conclusions: Our findings confirmed a high frequency of *KIT* and *NRAS* mutations in SUM, as well as a low incidence of *BRAF* mutations. We reported novel *KRAS*, *CTNNB1*, *TP53*, *ERBB2*, and *SMAD4* mutations in SUM. Our findings provide new insights into the molecular pathogenesis of SUM.

## INTRODUCTION

Across all human cancers, cutaneous malignant melanoma (MM) genome has one of the highest prevalence of somatic mutations. The type of mutations found is known to result from ultraviolet radiation (UVR) induced DNA damage [1]. The most commonly reported mutations in cutaneous malignant melanomas are located in *BRAF* and *NRAS* genes [2–5]. *BRAF* mutations are found in approximately 50% (22% to 70%) of cutaneous MM [3, 6, 7]. These mutations are more commonly detected in melanomas developing in the skin with intermittent sun exposure (non-chronic sun-damaged) - such as a trunk; than in non-UVR exposed skin surface. The most abundant *BRAF* mutation is associated with V600E amino acid substitution, and it comprises about 90% of all *BRAF* mutations found in MM. At the same time, *NRAS* mutations are detected in approximately 20% of MM and are more commonly reported in melanomas developing in the skin with chronic sun exposure [3, 6, 8]. The most common missense substitutions in *NRAS* are Q61R and Q61K [3].

Different patterns of genetic alterations, including type and frequency of mutations and chromosomal aberrations, is expected for melanoma subtypes [9]. Genomic profile differs not only between histopathological melanoma subtypes but also between melanomas developing in different anatomical locations [10]. It has been suggested that different melanoma subtypes, in fact, develop as a result of deregulation of type-specific molecular pathways and their spectrum is correlated with the degree of UVR exposure-related mutagenesis [9, 11]. On sun-exposed skin where the rate of cumulative solar damage is high, lentigo maligna and desmoplastic melanoma are observed. Superficial spreading melanoma is connected with low cumulative solar damage, and acral melanomas are described as nonsolar [12].

In the Cancer Genome Atlas study with whole-genome sequencing molecular profile of 333 non-UVR-related melanomas was described. It was shown that structural variants (deletions, duplications, tandem duplications, and foldback inversions) are significantly more common in non-UVR-related melanomas. Occurrence in some studies UVR-related mutational profile in acral sites suggests that nail plate and thick strata corneum do not provide complete UVR protection [11]. An increased number of genetic aberrations may be associated with a poorer prognosis [13].

The current 11. WHO Classification of Skin Tumours recognizes the most common acral melanoma histotype is acral lentiginous melanoma (ALM), followed by nodular cutaneous melanoma (NM) and superficial spreading melanoma (SSM) [12]. The majority of SSM and NM harbor mostly *BRAF* (50%) or *NRAS* (17%) mutations. On the contrary, in ALM, *BRAF* mutation is found in 17% of cases and *NRAS* - in 15% [6, 14]. On the other hand, ALMs harbor more often *KIT* mutations than other MMs – in 15% to 40% [6, 14]. ALM subtype was suggested to harbor specific gene amplifications and deletions resulting from unique genomic instability [15]. ALM is not correlated with sunburn or higher UVR exposure as it is developing in the skin unexposed to the sun, but chronic physical stress or pressure to acral locations may be a predisposing factor [8, 16–18].

Cutaneous MM located on the acral part of extremities - hand and foot melanoma (HFM) - comprises a rare group within all melanomas in Caucasians. Whole-genome sequencing study shown that structural changes and mutational signature of acral melanomas were dominated by different than other MMs sites. Acral melanoma presents unknown, non or lower UVR related, etiology [9, 19]. *BRAF*, *NRAS,* or *NF1* is mutated in HFM only in 42–55% of cases [19]. *KIT* mutation is detected in 3–40% of acral melanomas [19, 20]. Mutations in the promoter of *TERT* occur in 9–41% of HFM and are currently recognized as a prognostic factor [19]. *TERT* inhibitors are potentially available for use in the clinic. Others mutations observed in acral melanomas are *PAK1, CDK4, CCND1, CDKN2A, PTEN, P16, MAP2K2, ARID2, MITF, TP53, RAC1*, *RB1, SPRED1 ALK, ROS1, RET* and *NTRK1* [19]. Subungual melanoma (SUM) is a subgroup of HFM that arises from structures within the nail apparatus. SUM is exceedingly rare, representing 0.7–3.5% of all MMs in Caucasian populations, with an annual incidence of approximately 0.1 per 100 000 [6, 13, 21–23]. These tumors develop mostly on great toe and thumb. SUM seems to be not related to sun exposure, however, in Australian Melanoma Genome Project UVR signatures on acral melanomas occurred most frequently in subungual parts [11]. Both HFM and SUM seem to have poorer survival outcomes comparing to MMs of the other sites, with 5-year overall survival (OS) in the range 20–60% [22–27], although ‘Lieberherr’s in meta-analysis reports 77% [28]. Poor survival rates are in part as a result of delayed diagnosis since frequent misdiagnosis. In the literature, SUM present the most diverse mutation profile, including oncogenes like *NRAS, PIK3CA, EGFR, FGFR3, PTPN11, IDH2, ALK* and suppressor genes *STK11, TP53, APC* and high frequency of copy number aberrations in CCND1 and CDK4 than other MMs [13, 29]. The *BRAF* mutations occur less frequent in SUM compared to the MMs of other sites.

Due to limited data, the genetic profile of SUM is still under investigation. It is important to know tumor genetic profile as genetic mutations have a fundamental impact on the selection of targeted therapy such as BRAF/MEK inhibitors in *BRAF*-mutated tumors, tyrosine kinase inhibitors in KIT-mutated tumors and MEK inhibitors in *NRAS*-mutated tumors [6]. Generally, HFMs are described only by few, heterogeneous studies with various outcomes. SUM is a subgroup of HFM, so there is even fewer data. As next-generation sequencing (NGS, including whole genome/exome sequencing and gene panels) is now more widely used to characterize the genetic profiles of tumors, it was therefore selected as a basic research method for the project. Our study aimed to define the molecular profile of highly selected melanoma population using results from NGS panel of 50 cancer-related genes in homogenous cohort of patients with SUM.

## RESULTS

Thirty one Caucasian patients diagnosed with SUM were enrolled - 18 women and 13 men. The mean age was 62 years. Median Breslow thickness was 5 mm, and ulceration was observed in 74% of cases. In all cases, SLNB was performed with a positive result in 42% of SUM. The most common histological subtype diagnosed was ALM (76% of patients). Lower extremity digits involved 19 of 31 (61%) cases. The clinicopathologic characteristics of enrolled patients are presented in Table 1.

**Table 1:** Clinicopathologic characteristics of enrolled patients

Characteristics	Total *n* = 31
Age at diagnosis, years	
Median	66.24
Mean	62.05
IQR	15.12
Sex, *n* (%)	
Male	13 (42)
Female	18 (58)
SUM location, *n* (%)	
Hands	12 (39)
Feet	19 (61)
Primary tumor thickness (mm)	
Median	5
Mean	5.62
Ulceration, *n* (%)	
Absent	8 (26)
Present	23 (74)
Histopathological subtype, *n* (%)	
NM	5 (20)
SSM	0 (0)
ALM	19 (76)
LMM	1 (4)
NA	6
SLNB	
Positive, *n* (%)	13 (42)
Negative, *n* (%)	18 (58)
Median survival, mo (range)	42 (3,5–173)
Alive, *n* (%)	11 (35)
Dead, *n* (%)	20 (65)

Abbreviations: mm – millimeter, mo – month, n – number, y – year, NM – nodular melanoma, SSM – superficial spreading melanoma, ALM - acral lentiginous melanoma, LMM – lentigo malignant melanoma, NA – not available.

At least one mutation was detected in 12 patients (39%) with the most common mutations in the *KIT* gene in 5 cases (13%, two mutations in one case) and *NRAS* - in 4 cases (13%). *KRAS* mutation was detected in 2 patients, while other mutations in *CTNNB1, TP53, ERBB2, SMAD4, BRAF* – in single cases. The detailed summary of detected mutations with details is presented in Table 2.

**Table 2:** Summary of detected mutations

Patient	Age at diagnosis	Sex	Gene	Nucleotide change	Amino acid change	Location	Histopathologic type	Breslow thickness (mm)	SLNB	Ulceration	Survival status
1	69	F	*KRAS*	c.35G>T	p.G12V	foot	NM	5	Negative	Yes	Dead
2	78	M	*KIT*	c.1721_1729delCACAACTTC	p.Gln575_Pro577del	hand	ALM	1	Positive	No	Dead
3	60	F	*NRAS*	c.34G>T	p.G12C	foot	ALM	13	Positive	Yes	Dead
4	74	F	*NRAS*	c.38G>A	p.G13D	foot	ALM	20	Negative	Yes	Dead
5	49	F	*NRAS*	c.182A>G	p.Q61R	hand	NA	1	Negative	Yes	Dead
6	51	F	*SMAD4*	c.1081C>T	p.R361C	hand	ALM	4	Negative	Yes	Alive
			*KRAS*	c.35G>A	p.G12D						
7	52	M	*KIT*	c.1727T>C	p.L576P	hand	ALM	7	Positive	Yes	Dead
8	66	F	*KIT*	c.1727T>C	p.L576P	hand	ALM	6	Negative	Yes	Dead
			*KIT*	c.1676T>A	p.V559D						
9	64	M	*ERBB2*	c.2329G>C	p.V777L	hand	NM	14	Positive	Yes	Dead
10	49	F	*NRAS*	c.181C>A	p.Q61K	hand	NA	1	Negative	Yes	Dead
			*CTNNB1*	c.134C>T	p.S45F						
			*TP53*	c.797G>A	p.G266E						
11	64	M	*KIT*	c.1733_1735delATG	p.Asp579del	foot	ALM	9	Positive	Yes	Dead
12	26	F	*BRAF*	c.1799T>A	p.V600E	foot	NA	2	Negative	No	Alive

Abbreviations: F – female, M – male, NM – nodular melanoma, ALM - acral lentiginous melanoma, NA – not available, SLNB – sentinel lymph node biopsy.

## DISCUSSION

The mean patients age in our study was 62 years, which was consistent with 57.8–69 years reported of SUM patients in the literature [13, 21]. Comparing to other studies of acral location where one third of cases had no mutations detected; in our SUM group, no mutation was detected in even more cases (61%) [5]. In our cohort, median Breslow thickness was 5 mm, which is consistent with the literature of SUM [21]. The ulceration in our group was common (74%) which is also consistent with previous studies [21]. In our cohort, 76% of cases were identified as ALM, while in the study of 13 patients with SUM, including seven Caucasians, percentage was higher (85%). Another SUM study of 54 patients reports ALM in 65% of cases. The positive status of SLNB involved 42% of SUM patients, while in the scarce literature, the reported percentage is lower (31%) [21].

### KIT

The *KIT* gene encodes a transmembrane tyrosine kinase receptor, which is associated with activation of mitogen-activated protein kinase (MAPK) and phosphatidylinositol 3 kinase (PI3K/Akt) pathways [6]. MAPK and PI3K/Akt pathways play a role in the melanoma genesis because they regulate different cellular functions (proliferation, differentiation, and survival).

In our study, 5 (13%) SUM harbored *KIT* mutations. The identified *KIT* mutations included three single amino acid changes [L576P (*n* = 2) and p.V559D] and two deletions [p.Gln575_Pro577del, and p.Asp579del]. All of them were previously observed in melanomas [6]. In Reily’s study of 19 SUM cases, *KIT* mutation occurred in 11% of samples [21]. Although other studies of SUM present a higher percentage of 30–50% [25, 32] or no detected among 13 SUM patients [29]. In HFM/ALM, *KIT* mutations are frequent - occurring in 8.5–36% of tumors usually as a substitution of a single amino acid in exon 11, 13, or 17 [6, 8–10, 30–34]. Siroy et al. sequenced 54 patients with advanced acral melanoma and reported *KIT* mutation in 11% of cases [5]. Otherwise, the most common mutations were *NRAS* (24%), *BRAF* (19%), and *TP53* (6%). In Gong et al. meta-analysis incidence of *KIT* mutations in melanoma was higher in older patients and was associated with chronic sun-damage, acral and mucosal melanomas [35]. Mutations in *KIT* are relatively uncommon in MMs overall, about 1–6% [9, 31].

### NRAS

The *RAS* family (*N- K- H- RAS*) is a family of small proteins that provides to transduce signals triggered by extracellular growth factors. *NRAS* mutation is associated with activation of both MAPK and the PI3K/Akt pathways and is found in about 12–20% of all MMs [6, 7, 15]. Both MAPK and PI3K/Akt pathways are involved in the proliferation, differentiation, and survival of the cell [8].

In our study in 13% of cases were found *NRAS* mutation. The identified *NRAS* mutations included p.G12C, p.G12D, p.G13D, p.Q61K, and p.Q61R amino acid changes. All of them were previously observed in melanomas. Moreover, we have found that *BRAF* and *NRAS* were mutually exclusive and occurred only in tumors that were negative for *KIT* mutations, as reported in the literature [5, 31]. In Dica et al. study of 13 SUM patients, 30.8% presented *NRAS* mutation [25]. There is no significant difference of *NRAS* mutation in SUM/HFM/ALM compared to melanomas of the non-acral skin, it is detected in 7.5–25% of acral MM cases [2, 3, 5, 6, 8, 9, 29, 31, 32, 36]. *NRAS*+ melanomas are correlated with lower tumor-infiltrating lymphocytes (TIL) grade comparing to wild type, the anatomic site different than scalp or neck, and the presence of higher mitotic index. Lower TIL grade suggests a more immunosuppressed microenvironment, which can affect response to immunotherapy. The same study shows that *NRAS+* nor *BRAF+* was associated with overall melanoma-specific survival, but the risk of death was significantly more reduced for higher-risk *NRAS+* or *BRAF+* (T2 or higher stage) [2].

### KRAS

KRAS (Kristen Rat sarcoma), one of the RAS isoforms, plays an important role in human cancers acting upstream of BRAF [37]. Mutation in *KRAS* was detected in two (6%) of SUM tumors. The identified *KRAS* mutations included p.G12V and p.G12D amino acid changes. Both of them were previously observed in melanomas.

*KRAS* in MMs is infrequently mutated, although it is one of the most frequently mutated proto-oncogenes in human cancers. Siroy et al. identified *KRAS* mutation in 7.7% advanced MMs [5], and in the Catalogue of Somatic Mutations in Cancer [38] it has been reported in 2% of acral MMs. Also, in Min et al. study of MMs *KRAS* mutation was reported in 2.3% of cases [7]. There is no information about the present of *KRAS* mutation in SUM in the literature. *KRAS* is considered as having a rather weak oncogenic effect in MM [36]. In the study of the genetic profile of melanoma brain metastases, *KRAS* mutations were associated with reduced overall survival from brain metastasis resection [39].

### BRAF

*BRAF* mutation is associated with activation of the MAPK pathway, which frequently plays a role in human cancers. It is identified as less frequently in MMs on sun-protected skin (acral sites, mucosal). It was found in high frequencies in melanocytic naevi, which suggests that this somatic alternation occurs early in melanoma genesis [34]. In our material, only one patient with SUM was *BRAF*+ (3%), which is lower than previously reported 6.25–14.4% in SUM cases [21, 25, 29]. The identified most common *BRAF* mutation is a c. 1799 T>A transversion in exon 15, which causes p.V600E amino acid substitution, the most common mutation observed in melanomas [3]. Siroy et al. (2015) reported 2% of *BRAF* mutations in acral melanomas. In the literature of ALM, *BRAF* mutation is presented in 15–20% of cases [3, 6]. Compared to about 50% *BRAF* mutation in all primary and metastatic MMs, this mutation is under-represented in HFM/ALM [5, 33, 36, 40]. In the Swedish study, which evaluated 88 patients with primary ALM, it has been shown that *BRAF, NRAS,* and *KIT* mutations occur in ALM with a similar frequency of about 15%. *BRAF* mutations were significantly more common in younger patients, in females and in tumors located on the feet [6]. *BRAF+* melanomas are correlated with SSM subtype, and the presence of mitoses [2].

### CTNNB1

β-catenin (*CTNNB1* gene coding protein) is a part of the WNT pathway. It is fortifying cadherin-based adhesion at the plasma membrane and activating transcription in the nucleus [41]. Accumulation of cytoplasmic β-catenin promotes the transcription of proto-oncogenes and other various genes. One of the patients in our study had a mutation in *CTNNB1*. The identified *CTNNB1* mutation was p.S45F amino acid change, which was previously observed in MMs. There is no previously reported information in the literature of *CTNNB1* mutation in SUM. In Xu study of acral melanomas the positive expression of β-catenin was observed in 36 (72%) of patients, the expression of β-catenin, was not correlated with gender, age, or diseased body parts, but was significantly positively correlated with the tumor node metastasis stage and metastasis [42]. Shim et al. found a mutation in *CTNNB1* in acral melanomas in Korean patients [33]. In all MMs, *CTNNB1* mutation occurs in 2–5% of cases [43].

### TP53

Mutation in the p53 tumor suppressor gene has been linked to the majority of human cancers. It plays a role in a transcription activating target genes that mediate various functions (including deoxyribonucleic acid repaid, metabolism, apoptosis). Loss of wild-type p53 function, through mutation in p53 or alternation in pathway signaling, may promote cancer cell development, survival, and proliferation [44, 45]. One of our patients had a mutation in *TP53*. The identified *TP53* mutation included p.G266E amino acid change, previously observed in melanoma cell lines. Haugh et al. reported *TP53* mutation in 2 of 13 (15.4%) patients with SUM [29]. In the Catalogue of Somatic Mutations in Cancer [38] in acral MMs, the *TP53* mutation rate is about 9%, whereas Hayward et al. reported no acral melanomas had a mutation in *TP53* [9], and Yeh et al. found 1 case (0.8%) with *TP53* mutation [45]. In all sites, MMs mutation in *TP53* is presented at a low rate, occurring in 0–24% of MMs [9, 43].

### ERBB2

*ERBB2* encodes the HER2 receptor tyrosine kinase. Its alternations can lead to uncontrolled cell proliferation, consequently to oncogenesis, in various mechanisms. *ERBB2* mutation is uncommon in all types of MMs, a rate of about 1–3% [9]. In one case, we observed *ERBB2* mutation. The identified *ERBB1* mutation included p.V777L amino acid change. This mutation was not previously observed in melanomas. According to Gottesdiener et al., *ERBB2* mutation occurs in 2% in acral MMs, which is consistent with the rate in the Catalogue of Somatic Mutations in Cancer [38, 46]. However, there is no previous information about *ERBB2* mutation in SUM patients.

### SMAD4

The *SMAD4* takes part in transmitting chemical signals from the cell surface to the nucleus. It’s a part of transforming growth factor-beta (TGF-β) signaling pathway by activating SMAD proteins forming a complex with SMAD4 protein. As a central mediator of TGF-β signaling, *SMAD4* plays a role in cell differentiation, migration, invasion, and apoptosis [47]. Mouse models suggest that SMAD-mediated signaling in melanoma can play various functions, such as the proliferation of melanoma cells [48]. The loss of *SMAD4* can increase DNA instability by disturbing DNA damage response and mechanisms of repair [49]. The identified *SMAD4* mutation included p.R361C amino acid change. This mutation was not previously observed in melanomas.

The limitation of this study is a relatively small number of sequenced cases, but this limitation results from disease epidemiology. Another limitation is that used Cancer Hotspot Panel v2 includes only 50 cancer-related genes, so there may be existed loci not examined. For example, this panel does not include recently described human telomerase reverse transcriptase (*TERT*) promoter mutations, which were observed in approximately 9% of ALMs, and its amplification was correlated with poor outcome [50–52].

This is a first comprehensive analysis that focused on the genetic characterization of SUM in the Caucasian population. There is a lack of information in the literature about genetic profile in this subgroup of melanoma patients. Technological advances in molecular biology, particularly NGS, have increased the opportunities for mutation discovery in different subtypes of MM. We have presented the NGS results of 50 cancer-related genes of exceedingly rare among all MMs SUM cases in a homogenous group of Caucasians patients. Until now, our study is the largest of molecular profile in SUM Caucasian patients. In Reilly’s study of 54 patients with SUM in 19 of the molecular studies were performed with assessing a combination of one or more of *BRAF*, *c-KIT,* and *NRAS*, unfortunately, patients were with unknown racial background [21]. In Haugh’s study of SUM patients, *BRAF* mutation occurred in 1 in 13 samples [29]. Yeh et al. study of SUM *KIT* mutation was identified in 3 of 6 cases (more frequently than in acral MMs) [45]. Our findings confirmed previously reported in the literature of SUM amount of *KIT* and *NRAS* mutations, and we found a lower rate of *BRAF* mutations. Moreover, *KRAS*, *CTNNB1, TP53, ERBB2,* and *SMAD4* mutations not described previously in SUM appeared in our results.

The genetic profile of SUM is different from others sites of MMs, especially those with sun exposure. The most common mutations in our study were *KIT* (13%) and *NRAS* (13%) with coherent percentage comparing to previous literature. It stays in concordance with the previous studies that show that the nail plate can block UVB and UVB [9, 11, 53]. It leads to a non-UV-related malignancy pathway.

We have confirmed that SUM arises due to mutations in genes critical for differentiation (*KIT, NRAS*), cell cycle, and proliferation (*KIT, NRAS, KRAS, TP53, ERBB2, SMAD4*) and apoptosis (*TP53*).

Our study offers new insights into the genetics SUM subtype, for understanding pathogenesis and providing potential biomarkers for future studies. Molecular testing is now widely used in patients with advanced melanoma in the process of therapeutic decisions. Mutations reported in melanoma cells provide starting points for the development of the rational design of novel therapies, including immunotherapy agents. They also may provide to find the molecular pathogenesis and natural history of subtypes of this heterogeneous disease. We confirmed that SUM have different than other cutaneous melanomas genetic profile, which due to its rareness and lack of studies should be subjected to further analyzes in multicenter studies.

## MATERIALS AND METHODS

**Figure 1:**
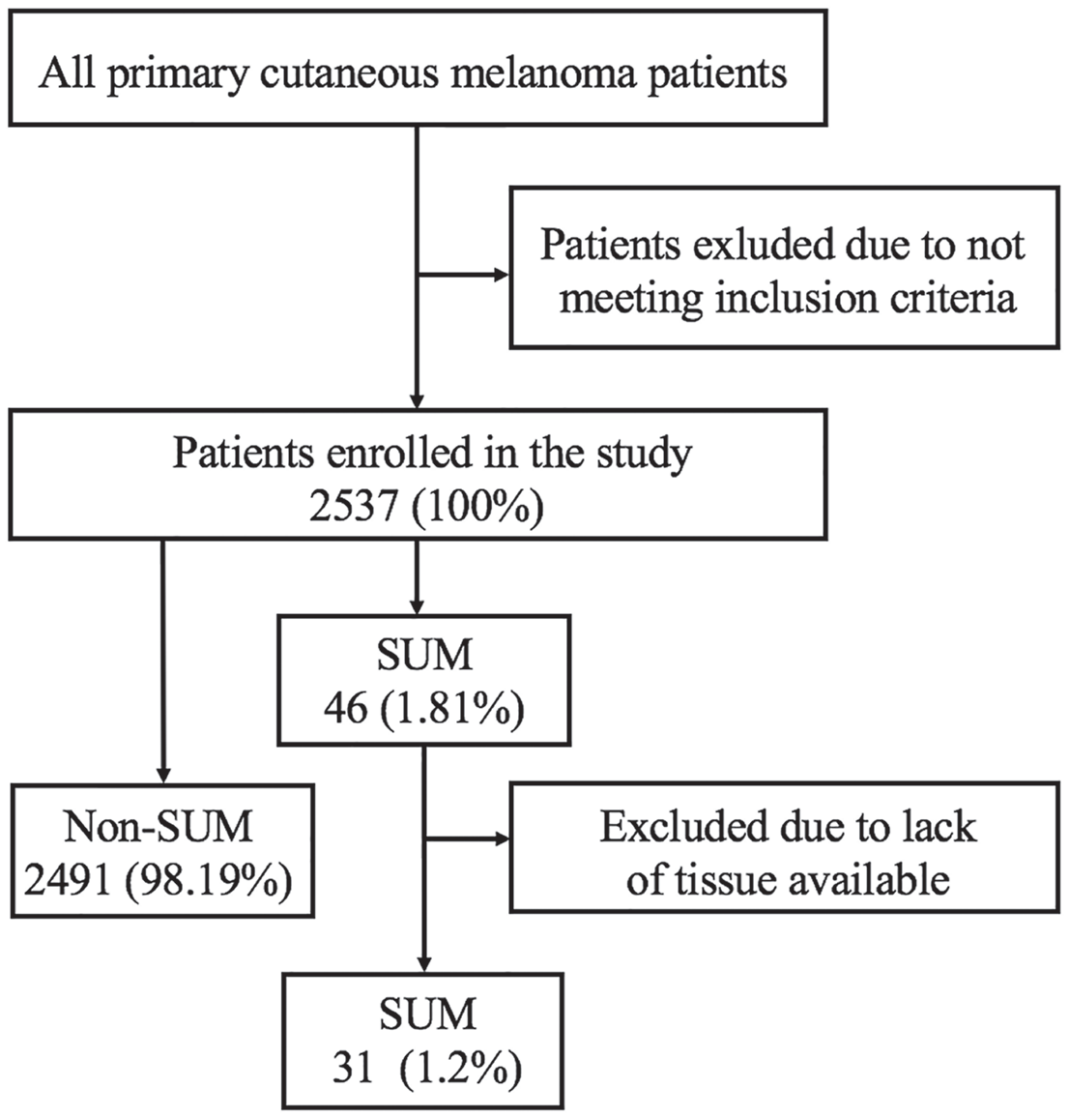
Data extraction. SUM - subungual melanoma.

Two thousand five hundred thirty-seven melanoma consecutive cases diagnosed and treated in Maria Sklodowska-Curie National Research Institute of Oncology, Warsaw, Poland, between 01. Jan 1997, and 31. Dec 2014, who underwent sentinel node biopsy, were screened, and 46 SUM patients were selected for the study (Figure 1). For screened MM population inclusion criteria were: diagnosis of primary cutaneous melanoma after excisional biopsy, Breslow thickness ≥ 0.75 mm or presence of ulceration, sentinel lymph node biopsy performed; while exclusion criteria included: a metastatic disease at the time of diagnosis, clinically palpable lymph nodes, incomplete medical records, or lack of primary tumor sample. We have included in this analysis only patients in clinical stage I-II undergoing sentinel node biopsy to constitute the homogenous population, in thirty one cases the high quality pathological specimens were available for molecular analyses for study purposes. Informed consent from all patients was obtained.

### Next-generation sequencing

SUM DNA was sequenced by the Ion Proton sequencing platform using the Ion AmpliSeq Cancer Hotspot Panel v2 to identify frequently mutated regions in 50 cancer-related genes including *ABL1, EZH2, JAK3, PTEN, ACT1, FBXW7, IDH2, PTPN11, ALK, FGFR1, KDR, RB1, APC, FGFR2, KIT, RET, ATM, FGFR3, KRAS, SMAD4, BRAF, FLT3, MET, SMARCB1, CDH1, GNA11, MLH1, SMO, CDKN2A, GNAS, MPL, SRC, CSF1R, GNAQ, NOTCH1, STK11, CTNNB1, HNF1A, NPM1, TP53, EGFR, HRAS, NRAS, VHL, ERBB2, IDH1, PDGFR, ERBB4, JAK2, PIK3CA*.

DNA concentration was measured using Qubit fluorimeter and dsDNA HS Assay Kit (Thermo Fisher Scientific). The libraries were prepared using the Ion AmpliSeq™ Library Kit 2.0, Ion AmpliSeq™ Cancer Hotspot Panel v2 (Thermo Fisher Scientific), and the Ion Xpress Barcode Adapters Kit, according to the manufacturer’s instructions (Thermo Fisher Scientific).

The prepared libraries were subjected to double purification using Agencourt AMPure XP (Beckman Coulter Genomics) according to the manufacturer’s instructions (Ion AmpliSeq Library preparation - Thermo Fisher Scientific). Ion Library Quantitation Kit (Thermo Fisher Scientific) was used for real-time PCR analysis. Sequencing was performed on the Ion S5 sequencer (Thermo Fisher Scientific) using the Ion 520™ & Ion 530™ Kit-Chef kit according to the manufacturer’s instructions.

### Data analysis

The raw data generated during the sequencing was processed using Torrent Server Suite 5.2 (LT). The obtained sequences were mapped to the reference sequence of the human genome (hg19). Various variants (SNPs, mutations) were detected using Variant Caller v5.2, part of Torrent Server Suite 5.2. The manufacturer’s default parameters for AmpliSeq somatic were used: minimum allele frequency - SNP = 0.018 / INDEL = 0.02, minimum quality - 6, minimum coverage - 100. The called mutations were reviewed using IGV - Integrative Genomics Viewer (Broad Institute). In addition, Torrent Server Suite 5.2 generated FASTQ files that were used for analysis using other software: Biomedic Genomic Workbench 4.0 (QIAGEN) and GALAXY (http://wannovar.wglab.org/). The following default parameters used in the analysis using: Biomedical Genomic Workbench 4.0 (minimum allele frequency - 0.05, minimum quality -10, minimum coverage – 100) and for GALAXY (minimum allele frequency - 0.05, minimum quality -15, minimum coverage – 100). The wANNOVAR software (http://wannovar.wglab.org/) was used to annotate the variants called by TSS and GALAXY.

## Abbreviations

MM: malignant melanoma
UVR: ultraviolet radiation
ALM: acral lentiginous melanoma
NM: nodular cutaneous melanoma
SSM: superficial spreading melanoma
HFM: hand and foot melanoma
SUM: subungual melanoma
OS: overall survival
NGS: next-generation sequencing
MAPK: mitogen-activated protein kinase
PI3K/Akt: phosphatidylinositol 3 kinase
TIL: tumor-infiltrating lymphocytes
TGF-β: transforming growth factor-beta
